# The effects of pastoral intensification on the feeding interactions of generalist predators in streams

**DOI:** 10.1111/mec.14459

**Published:** 2017-12-23

**Authors:** C. E. Pearson, W. O. C. Symondson, E. L. Clare, S. J. Ormerod, E. Iparraguirre Bolaños, I. P. Vaughan

**Affiliations:** ^1^ Cardiff School of Biosciences Cardiff University Cardiff UK; ^2^ School of Biological and Chemical Sciences Queen Mary University London UK; ^3^ Department of Genetics, Physical Anthropology and Animal Physiology Faculty of Science and Technology University of the Basque Country Bilbao Spain

**Keywords:** agriculture, feeding interactions, freshwater, invertebrates, predators

## Abstract

Land‐use change can alter trophic interactions with wide‐ranging functional consequences, yet the consequences for aquatic food webs have been little studied. In part, this may reflect the challenges of resolving the diets of aquatic organisms using classical gut contents analysis, especially for soft‐bodied prey. We used next‐generation sequencing to resolve prey use in nearly 400 individuals of two predatory invertebrates (the Caddisfly, *Rhyacophila dorsalis,* and the Stonefly *Dinocras cephalotes*) in streams draining land with increasingly intensive livestock farming. *Rhyacophila dorsalis* occurred in all streams, whereas *D. cephalotes* was restricted to low intensities, allowing us to test whether: (i) apparent sensitivity to agriculture in the latter species reflects a more specialized diet and (ii) diet in *R. dorsalis* varied between sites with and without *D. cephalotes*. DNA was extracted from dissected gut contents, amplified without blocking probes and sequenced using Ion Torrent technology. Both predators were generalists, consuming 30 prey taxa with a preference for taxa that were abundant in all streams or that increased with intensification. Where both predators were present, their diets were nearly identical, and *R. dorsalis*'s diet was virtually unchanged in the absence of *D. cephalotes*. The loss of *D. cephalotes* from more intensive sites was probably due to physicochemical stressors, such as sedimentation, rather than to dietary specialization, although wider biotic factors (e.g., competition with other predatory taxa) could not be excluded. This study provides a uniquely detailed description of predator diets along a land‐use intensity gradient, offering new insights into how anthropogenic stressors affect stream communities.

## 1 INTRODUCTION

Anthropogenic activities are altering biodiversity and species composition at an unprecedented rate globally (Sala et al., 2000). The complex direct and indirect processes involved, including trophic links, competition and mutualism, mean that patterns of species loss can be difficult to predict, whilst changes in species composition can have unexpected consequences for ecosystem processes and dynamics (Holling, 1973; McCann, 2000). Examples of this complexity include patterns of secondary extinctions following species loss (Eklöf & Ebenman, 2006), and changes in ecological processes such as decomposition and community respiration triggered by species losses at different trophic levels (e.g., Atwood, Hammill, & Richardson, 2014). Predicting and mitigating the ecosystem‐level effects of anthropogenic stressors therefore requires an improved understanding of interspecific interactions.

The relative strengths of trophic links within food webs are fundamental to many ecosystem functions, governing transfers of energy and nutrients (Memmott et al., 2005). Changes in the abundance or feeding behaviour of consumers can result in wide‐ranging direct and indirect consequences for ecosystem functioning (McCann, 2000). Equally, changes in the abundance of basal resources or primary consumers can propagate up the food web, leading to species losses. Predators can be particularly vulnerable to perturbations as a result of their higher trophic positions, lower population densities and slower reproductive rates (Purvis, Gittleman, Cowlishaw, & Mace, 2000). In addition to a simple alleviation of top‐down control, reduction in predator populations can have complex effects on community structure due to high interconnectivity, intraguild predation and competition between predators (Finke & Denno, 2005; Petchey, 2004). Concomitantly, the perturbation may alter the feeding behaviour and prey choice of generalist consumers by changing prey abundance, the availability of refugia for prey and the competitive abilities of predators (Symondson, 2002). Identifying how predator diets respond to stress gradients could reveal thresholds at which ecosystem functioning may be disrupted (Woodward, 2009). In addition, comparing the trophic interactions of predatory species may explain their relative sensitivity to stressors, for example, whether more specialized foragers are less resistant to stress (Clavel, Julliard, & Devictor, 2011). Thus, consideration of predator–prey and predator–predator interactions, with analysis of prey choice, is essential when assessing the effects of stressors on communities (Gray et al., 2014; Woodward, 2009).

Streams are amongst the most sensitive ecosystems to anthropogenic disturbance, with the intensification of catchment land use a major driver of biodiversity loss (Dudgeon et al., 2006). Benthic macroinvertebrates dominate stream food webs in terms of abundance and number of interactions, and the community has key roles in a wide range of ecosystem processes (Covich, Palmer, & Crowl, 1999). Despite changes in invertebrate community structure, including predator populations, being widely reported as a consequence of land‐use intensification (e.g., Harding, Young, Hayes, Shearer, & Stark, 1999; Yuan & Norton, 2003), the associated modifications to stream food webs have received little attention (Gray et al., 2014). Several experimental studies have confirmed that changing predator densities produces complex effects on stream ecosystems (e.g., Rodríguez‐Lozano, Verkaik, Rieradevall, & Prat, 2015; Woodward, Papantoniou, Edwards, & Laurisden, 2008), but studies of changes in trophic interactions across stress gradients have been limited to acidification (Layer, Riede, Hildrew, & Woodward, 2010) and temperature (O'Gorman, Fitch, & Crowe, 2012). This dearth of studies may be due to the difficulties of resolving freshwater food webs: predator–prey interactions cannot be observed directly, whilst visual identification of predator gut contents is demanding and may overlook soft‐bodied prey (Symondson, 2002). Advances in molecular ecology (Symondson, 2002), including most recently next‐generation sequencing (NGS) (Pompanon et al., 2012), have made rapid and accurate determination of predator diets possible and offer great potential for assessing anthropogenic effects on food web structure (Clare et al., 2014). Approaches using NGS have proven to be successful with a range of vertebrate predators (e.g., Brown et al., 2014; Vesterinen, Lilley, Laine, & Wahlberg, 2013) and, recently, with terrestrial predators (e.g., Gomez‐Polo et al., 2015; Petráková et al., 2015; Piñol, San Andres, Clare, Mir, & Symondson, 2014; Tiede et al., 2016). Here, we extend this to freshwater macroinvertebrates for the first time by analysing the dietary choices of two dominant predators that are thought to have a pivotal role in freshwater food webs.

The goal of this study was therefore to use NGS to quantify the diet and prey preferences of two invertebrate generalist predators, *Rhyacophila dorsalis* (Trichoptera) and *Dinocras cephalotes* (Plecoptera), and assess how these properties changed along a gradient of agricultural intensification. By sampling in four seasons, we also aimed to assess annual variation in diet. We focused on the potential effects on streams of livestock production, primarily sheep rearing, which covers over >50% of the UK land surface (Morton et al., 2011). Stream catchments ranged from those containing semi‐natural vegetation and supporting low sheep densities, to catchments dominated by heavily fertilized pasture sown with non‐native grasses, grazed by much higher sheep densities. Based on a previous field study (C. E. Pearson, unpublished data), *R. dorsalis* and *D. cephalotes* were two of the region's most widespread aquatic predators, but showed contrasting distributions: *R. dorsalis* was abundant in all streams sampled, whereas *D. cephalotes* was absent from more intensive catchments. Our aims in this study were to identify how pastoral intensification and season affected the diets of both predators, and whether differences in their diets could account for their differing sensitivity to agricultural land use. It was predicted that: (i) being generalists, both predators would consume a wide range of prey taxa in proportion to their availability, so that their diets simply tracked changes in potential prey across the agricultural intensity gradient, but that (ii) the lower resilience of *D. cephalotes* to agricultural stressors compared to *R. dorsalis* would be reflected in narrower diet breadth and stronger prey selection; and (iii) the absence of competition from *D. cephalotes* at the highest agricultural intensities would result in *R. dorsalis* having a wider feeding niche relative to the available prey diversity.

## 2 METHODS

### 2.1 Sample collection and preparation

Ten upland streams in South Wales were selected to span a range of pastoral land‐use intensity. The catchments of all 10 were dominated by pastoral agriculture (>75% of catchment area) and had sandstone/mudstone geology, whilst the streams had similar base‐cation availability (Larsen, Ormerod, & Vaughan, 2009) and were matched as far as possible on the predominant substrate, depth, width and altitude. The catchments differed in the extent of unimproved pasture (unfertilized, with native grass species supporting low densities of livestock; 0%–100% cover) and improved pasture (fertilized and reseeded with high stocking densities; 0%–86% cover; JNCC, 2000). Agricultural intensity is difficult to quantify, being influenced by factors including stocking density and fertilizer applications for which high‐resolution data are difficult to obtain. Therefore, an index of in‐stream physicochemical conditions was used as a surrogate for agricultural intensity. The index (hereafter “intensity score”) was the first principal component from an analysis of 45 variables recorded at every site, which included water chemistry, channel morphology, bankside vegetation, erosion extent, flow velocity and sedimentation (see Appendix S1 for full details of the intensity score). Larger intensity scores equated to higher nutrient concentrations, greater poaching of the banks and fine sediment cover of the stream bed: all associated with intensive livestock production (Pearson, Ormerod, Symondson, & Vaughan, 2016).

Samples were collected in February, June, September and December 2013 to capture seasonal variation in abiotic conditions and prey populations. On each sampling occasion, three one‐minute kick samples were conducted to assess the abundance of potential prey, using a 1‐mm mesh size D‐frame net, covering all microhabitats in proportion to their abundance, and samples were preserved in 70% ethanol. Further kick samples were then performed to obtain *R. dorsalis* and *D. cephalotes* for molecular analysis. The first 10 individuals of each species, or as many as were found in one‐hour searching time, were immediately preserved in 100% ethanol in individual centrifuge tubes, giving a total of 497 individuals across all sampling periods.

In the laboratory, kick samples were rinsed through a 500‐μm sieve and macroinvertebrates were removed identified to genus, or a lower taxonomic resolution where this was not practicable, using traditional morphology and counted. The foregut of each predator was dissected into a sterile Eppendorf, for immediate extraction, excluding as much of the predator's own tissue as possible.

### 2.2 DNA extraction and Primer Selection

DNA was extracted from the dissected gut contents using the Qiagen blood and tissue kit (Qiagen, UK) per the manufacturer's instructions for animal tissue. Additionally, DNA was extracted from the legs of a wide range of potential prey and both predator species using the less costly “Salting out” method (Miller, Dykes, & Polesky, 1988) (Appendix S2). Negative controls were included alongside each batch of extractions to monitor for contamination (King, Read, Traugott, & Symondson, 2008). Extracted DNA was stored at −20°C prior to amplifications.

A single pair of general invertebrate primers was selected for amplification of predator gut contents; LCO‐1490 (5′‐GGTCAACAAATCATAAAGATATTGG‐3′) (Folmer, Black, Hoeh, Lutz, & Vrijenhoek, 1994) and HCO‐1777 (5′‐ACTTATATTGTTTATACGAGGGAA‐3′) (Brown, Jarman, & Symondson, 2012), which target a 287‐bp fragment of invertebrate CO1 genes. Blocking probes were not used as the phylogenetic proximity of predator and prey made it likely that a blocking probe would prevent amplification of many prey species (Piñol, Mir, Gomez‐Polo, & Agustí, 2015). These primers were tested to confirm their ability to amplify DNA from 18 invertebrate taxa (Appendix S2). Temperature gradient polymerase chain reactions (PCRs) were performed to determine the optimal annealing temperature. PCRs were run on a Peltier Thermal Cycler in 25 μl reaction volumes with conditions as follows: 1× buffer, 4 mm MgCl_2_, 0.05 mm dNTPs (Promega), 0.1 mm of each primer, 0.625 U Taq polymerase (Promega) and 2.5 μl of template DNA with an initial denaturation at 95°C for 2 min, 35 cycles of 30 s at 94°C, 30 s at 46°C, 45 s at 72°C and a final extension of 10 min at 72°C. Amplification success was determined by running 2 μl of each PCR product on a 2% aragose gel stained with EtBr. This primer pair was found to amplify all 18 of the tested taxa and was therefore used in further analysis.

### 2.3 Ion torrent sequencing

Predator gut content DNA samples were prepared for Ion Torrent sequencing following recommendations for unidirectional sequencing (Ion Amplicon Library Preparation, Fusion Method). Samples were processed and sequenced in two batches, samples collected in June and December (*n* = 218), and samples collected in February and September (*n* = 176) (Appendix S3). Three individuals were included in both sequencing runs to determine whether there were differences in sequencing outputs between the two runs.

Sixteen forward primers were designed, each consisting of ion torrent primer A, LCO‐1490 primer and a unique 10 base pair multiplex identifier sequence (MID). Fifteen reverse primers, each with the ion torrent primer B linked to the HCO‐1777 primer and a unique MID, were also designed. This gave 240 unique combinations of forward and reverse primer pairs, allowing each individual to be identified from the pooled data from each of two sequencing plates.

The DNA from predator gut extracts was amplified in 20‐μl reactions containing 2 μl of template DNA, 10 μl of Quiagen multiplex master mix, 6 μl of water and 1 μl of the specific forward and reverse primers (at 10 μm). The PCR was run as above with the initial denaturation extended to 15 min. The intensity of each gel electrophoresis band, as visualized on UVP VisionWorks^®^ ls analysis Software, was compared with the intensity of the 500‐bp ladder band, allowing all amplicons to be pooled into an equimolar library according to their intensity relative to the ladder. A gel extraction was performed on each pool to remove primer/dimer. Because of the high concentration of DNA in the pooled sample for the first round of sequencing (June and December), the sample was diluted 1:5 with purified water before running 20 μl in each of four lanes on a 1.5% aragose gel. In the second sequencing batch (February and September samples), 20 μl of the undiluted pooled sample was run in three gel lanes. The specific bands were dissected from the gel and processed using the QIAquick Gel Extraction kit (Qiagen) with a final elution volume of 40 μl. High‐throughput sequencing was conducted on an Ion Personal Genome Machine (IPM) using 400 bp chemistry at the Centre de Recerca en Agrigenòmica, Barcelona. In an attempt to account for the different number of individuals in the two sequencing runs and standardize the number of sequences per individual, a 318 chip (>3 million reads) was used for the first sequencing round and a 316 chip (>1.5 million reads) for the second.

### 2.4 Sequence analysis

Sequence processing was performed in Galaxy (usegalaxy.org, Blankenberg et al., 2010; Giardine et al., 2005; Goecks, Nekrutenko, Taylor, & Team, 2010). Sequences were split by forward and reverse MIDs and adaptors, primers and MIDs were removed before filtering sequences by length (260–300 bp). Sequences from each individual were collapsed into unique haplotypes, and rare haplotypes (<2 copies) were excluded. The remaining sequences from all individuals were combined and clustered into molecular operational taxonomic units (MOTU) using the *usearch* algorithm in Qiime (usearch61; Edgar, 2010). MOTU clustering was repeated with similarity thresholds decreasing in increments of 0.01 from 0.97 to 0.87. For each similarity value, representative sequences were selected from the resultant MOTUs and “BLASTed” directly at the NCBI website (http://blast.ncbi.nlm.nih.gov/blast) using nucleotide blast (Zhang, Schwartz, Wagner, & Miller, 2000) optimized for very similar sequences (megablast) on the nucleotide collection (nr/nt) using default parameters. The output from the blast alignment was imported into MEGAN (MEtaGenomics ANalyzer; Huson, Auch, Qi, & Schuster, 2007), which assigns taxonomy to each MOTU at the lowest level that encompasses the top blast hits. The optimal similarity threshold was the value that resulted in the lowest number of species (excluding chimeras) with multiple MOTUs allocated to them, whilst retaining the majority of species assignments (0.89).

### 2.5 Assigning taxonomy

The representative sequences from the optimal MOTU clustering were compared to the bold database (http://www.barcodinglife.org). Sequences were initially queried against the “species‐level barcode records” database. If a match was not found, then the sequence was queried against the “all barcode records” database, which includes barcodes that do not have species‐level identification. A sequence was assigned at the highest taxonomic resolution to which it had a >98% similarity (Clare, Lim, Fenton, & Hebert, 2011; King, Symondson, & Thomas, 2015). MOTUs producing no match (with >98% similarity) or matching to contaminants (e.g., bacteria, humans and algae) were removed from further analysis. The presence of each assigned MOTU was determined for each individual predator.

### 2.6 Data analysis

All analyses were carried out in R v. 3.1 (R Core Team, 2015). Analyses were based on the number of predator individuals testing positive for each prey species as NGS cannot reliably determine the relative abundance or biomass of prey species consumed (Pompanon et al., 2012).

Combining the data from two NGS runs may have introduced additional variation, as runs can vary due to factors such as sample storage time, or more likely slight differences between batches of reagents. To avoid any problems, we employed a conservative strategy in which: (i) in addition to running our analyses using all four seasons, we also ran each analysis separately for the two sampling runs (results not shown), confirming that the results were consistent with the overall analysis, and (ii) where we tested for differences amongst seasons in a model, we only compared seasons from the same NGS run (i.e., June vs. December and February vs. September).

### 2.7 Changes in potential prey resources across the intensity gradient

Changes in the macroinvertebrate community across the intensity gradient were visualized using nonmetric multidimensional scaling (NMDS) in two dimensions using Bray–Curtis similarities (Bray & Curtis, 1957). Data from all kick samples within each site were combined and fourth‐root transformed prior to analysis to down‐weight the influence of the most abundant taxa (Clarke & Warwick, 2001). The correlation between NMDS site scores and the agricultural intensity score was assessed with a permutation test in the vegan package's envfit function (Oksanen et al., 2013).

Changes in prey abundance, richness and rarefied richness across the intensity gradient were modelled against agricultural intensity using linear mixed effects models (LMMs) in R's nlme package (Pinheiro, Bates, DebRoy, Sarkar, & R Core Team, 2015). Site was included as a random factor to account for the nonindependence of four seasonal samples taken from each location. Sampling completeness for predator diets was assessed by constructing smoothed species accumulation curves using vegan's *specaccum* function (Oksanen et al., 2013), and estimating the total number of species present, as well as the number of individual predators needed to find 90% and 50% of the total prey taxa.

### 2.8 Comparison of *D. cephalotes* and *R. dorsalis* diets

The diets of *D. cephalotes* and *R. dorsalis* were compared in terms of their overall composition, breadth, overlap and their apparent prey choices. The overall similarity amongst sites and seasons (*n* = 59) was assessed using NMDS as described above, with the proportion of individuals that consumed each prey taxon in place of prey abundance. For sites and seasons where both predators were present (*n* = 21), permutational multivariate analysis of variance was used to test whether their diets were significantly different (vegan's adonis function; Oksanen et al., 2013). The two species' diet breadths were compared for the same 21 site‐season combinations using: (i) the mean number of prey taxa detected per individual, and (ii) Levins' standardized measure of niche breadth (*B*_*A*_; equation 1 in Razgour et al., 2011), where smaller values of *B*_*A*_ indicate greater specialization. *B*_*A*_ controls for the number of potential prey, facilitating comparisons amongst locations. Differences in the breadth measures between the predators were tested using LMMs with the site as a random term to control for multiple, seasonal samples. To test whether the diet breadth of *R*. *dorsalis* increased in the absence of *D. cephalotes*,* B*_*A*_ for *R. dorsalis* was modelled across all sites using a LMM, with the presence–absence of *D. cephalotes* as an explanatory variable and site as a random term.

Dietary overlap between the predators was assessed using Pianka's (1973) measure of resource sharing. Observed diets were compared to diets generated with null models to test whether niche overlap was greater than expected by chance. Using the ecosimr package (Gotelli, Hart, & Ellison, 2015), 10,000 Monte Carlo simulations were performed to generate randomized utilization matrices for the two predators. Pianka's measure was applied to these random matrices and the results compared to the observed diet matrix. The proportion of simulated matrices exceeded by the observed data gave the probability that the overlap was greater than was expected at random (Gotelli and Ellison, 2015).

Finally, the observed frequencies of different prey species in the diets of *R. dorsalis* and *D. cephalotes* were compared to those expected under the null model of Agustì et al. (2003) to test for evidence of prey selection. The model assumes that prey species are consumed in proportion to their relative abundances by predators, and by comparing the observed frequencies with which prey species were consumed to the frequencies under the null model, indicates whether a predator disproportionally selects a species (higher observed than modelled frequency) or avoids a species (lower than modelled; Agustì et al., 2003). Data were pooled across seasons at the site level and prey abundance estimated from the kick samples. The null model was run for 10,000 iterations to allow 95% confidence limits to be generated around the modelled frequency of each prey species (Davey et al., 2013).

The overall strength of prey selection by each predator was summarized by dividing the absolute differences between the observed and expected consumption frequency of each taxon by the total number of prey consumed, and then summing the differences across all taxa in the diet. The resulting measure equals zero when observed and expected values are identical, and reaches one when there is no overlap between the observed and expected patterns of consumption. An LMM was used to test whether *R. dorsalis* became less selective in its prey choice in the absence of *D. cephalotes*. The presence/absence of *D. cephalotes* was a fixed effect in the model and site a random term.

### 2.9 Changes in diet with increasing agricultural intensity

The overall effect of increasing agricultural intensity on predator diet focused upon *R. dorsalis* as it occurred across the complete gradient. The mean number of prey taxa, dietary specialization (*B*_*A*_) and overall prey selectivity were modelled across the 40 site–season combinations as a function of the agricultural intensity score and season using LMMs. Site was modelled as a random term to account for the nonindependence of four seasonal samples from each location, and the interaction between season and agricultural intensity score was included to determine whether land‐use effects varied by season. For each of these three models, the model structure was determined by selecting the model with the lowest AIC value from amongst the four models representing every possible combination of predictor variables (Burnham & Anderson, 2004).

## 3 RESULTS

### 3.1 Sequences analysis

DNA was successfully sequenced from the gut contents of 394 individuals (79%); 237 *R. dorsalis* and 157 *D. cephalotes* (Appendix S3). The two sampling rounds recovered 5.3 and 3.2 million sequences, respectively, of which 1.13 and 1.08 million remained after sequence processing. Using a 0.89 similarity cut‐off, sequences were assigned to 73 MOTUs in the first sequencing batch (June and December samples) and 78 MOTUs in the second (February and September samples). After removal of contaminants (nearest similarity was identified as a nonprey item, e.g., human, bacterium or freshwater mould) and MOTUs without a match at 98% similarity, 43 MOTU remained from the first sequencing round and 51 from the second. Where necessary, MOTUs were combined to the taxonomic level identified in the kick samples to ensure consistency across analyses. Of the sequences assigned to MOTUs, predator DNA accounted for 3.14% (3.50% in *R. dorsalis* and 0.32% in *D. cephalotes*) with similar frequencies in the two sequencing rounds. There was also the occurrence of intraguild predation with 10% of *R. dorsalis* individuals consuming *D. cephalotes* and 27% of *D. cephalotes* consuming *R. dorsalis*.

**Figure 1:**
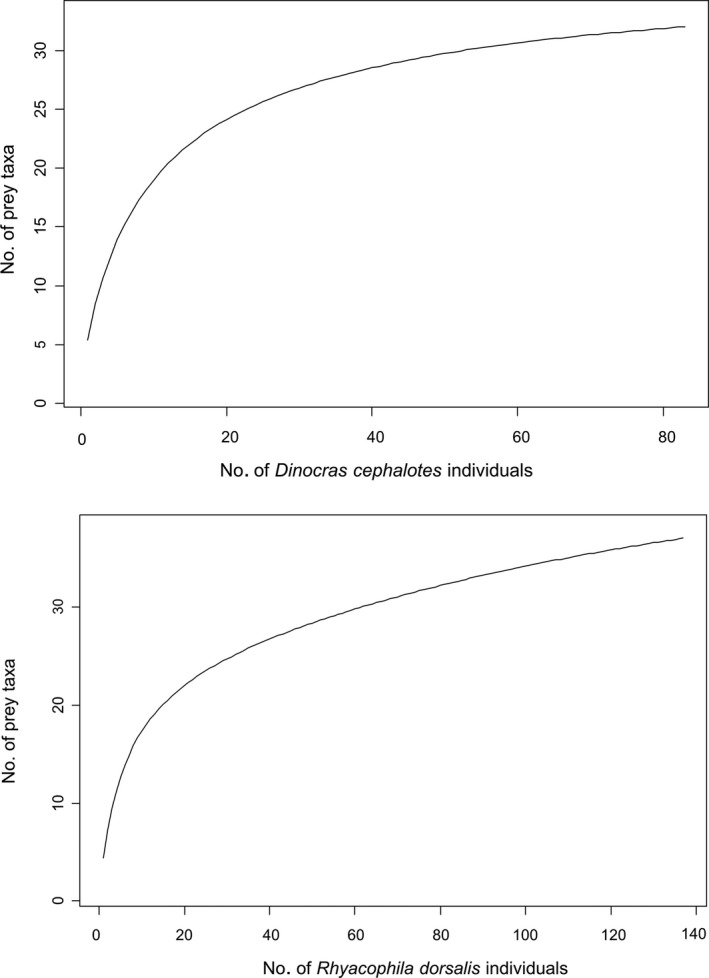
Smoothed yield‐effort accumulation curves for the number of prey taxa consumed by the two predators as a function of individual predators analysed

Sampling completeness of both predators' diets was high, with 90% of prey taxa being detected after 61 and 67 individuals being sampled for *D. cephalotes* and *R. dorsalis* respectively (Figure 1). Fifty per cent coverage was reached with just eight and nine individuals. The estimated total number of prey taxa was around 25 for both predators (Figure 1).

### 3.2 Distribution of predators and prey resources

**Figure 2:**
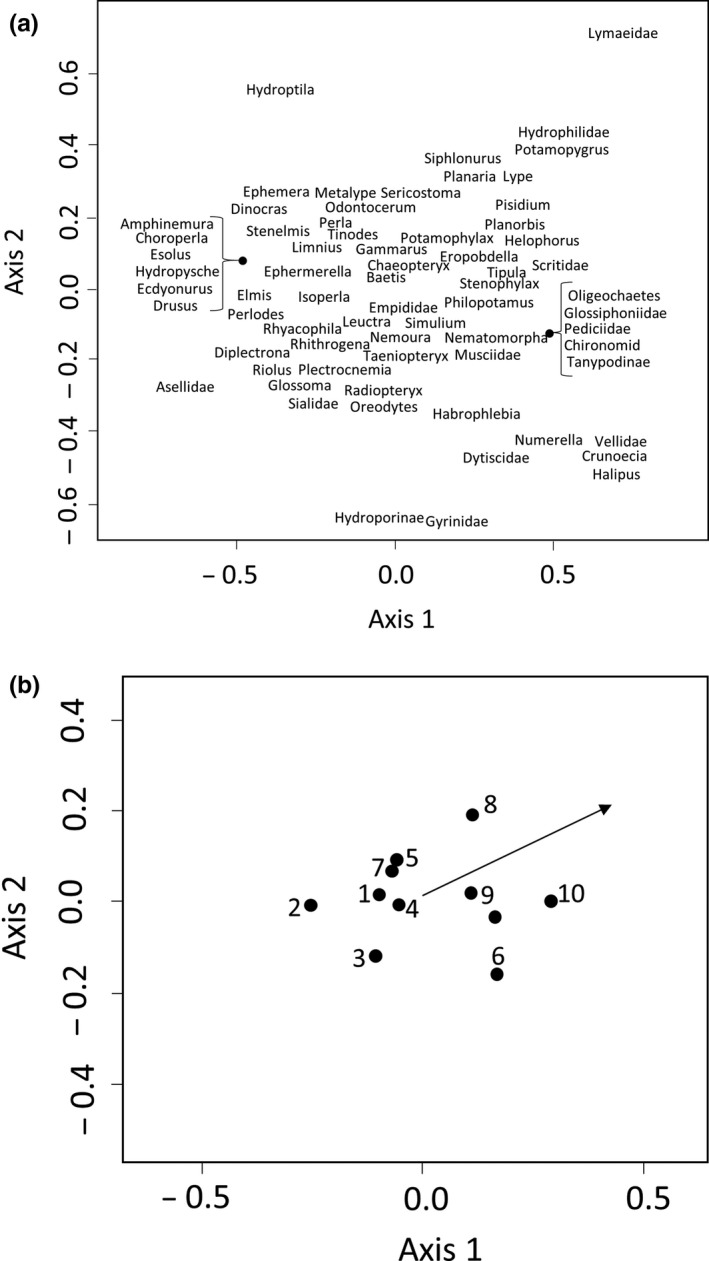
Nonmetric multidimensional scaling analysis of prey community composition across the 10 streams spanning a gradient of agricultural intensity. Panel A shows the “species” scores, whilst panel B plots the individual sites, numbered based on rank of agricultural intensity (1 lowest, 10 highest). The arrow on the right‐hand plot shows the vector of increasing intensity score (*r* = .85 *p* = .002)

*Rhyacophila dorsalis* was present in all streams, whereas *D. cephalotes* was absent from the four most intensively farmed sites, where nitrate was >8 mg/L and there was >13 mg/L resuspendable inorganic sediment (see Appendix S1 for details of the environmental variables). The invertebrate community changed with increasing intensity from communities dominated by Ephemoptera, Plecoptera, Trichoptera and Elmidae to communities dominated by molluscs and dipteran larvae in sites with high pastoral intensity (Figure 2; permutation test *p = *.002). Invertebrate abundance and richness were not significantly related to agricultural intensity across the 2013 samples (*F* = 0.22, *p = *.65 and *F* = 0.41, *p = *.55) although there was a nonsignificant decline in rarefied richness (*F* = 4.24, *p = *.07), which was significant across a larger set of streams (C. E. Pearson, 2012 unpublished data).

### 3.3 Comparison of *D. cephalotes* and *R. dorsalis* diet

**Figure 3:**
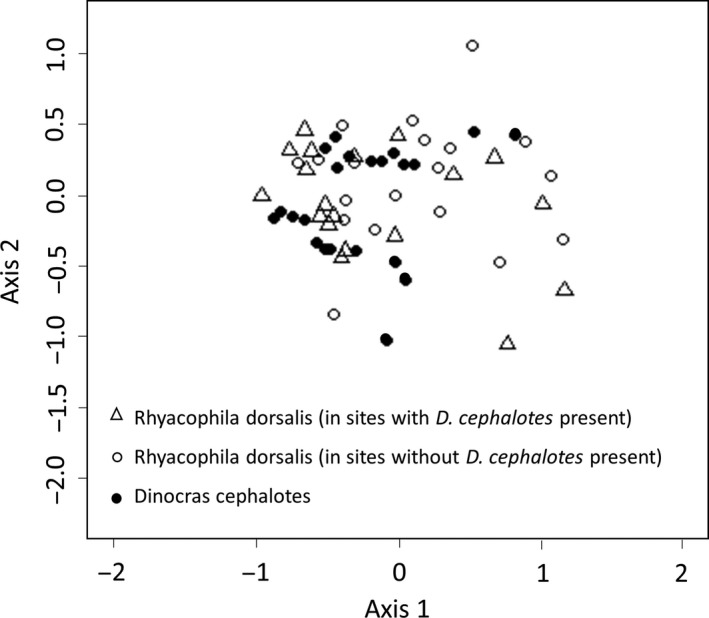
Results of nonmetric multidimensional scaling analysis of diet composition for predators *Rhyacophila dorsalis* and *Dinocras cephalotes*. Ordination results of each site, based on the number of predator individuals consuming each prey taxa

**Figure 4:**
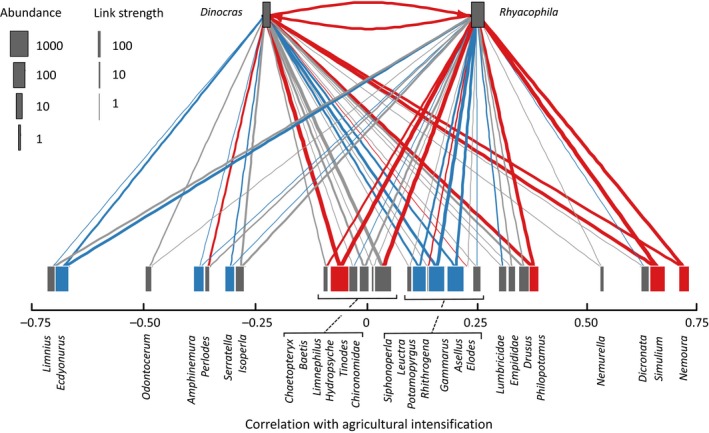
The complete set of trophic links across the 10 streams, including intraguild predation, revealed by next‐generation sequencing. Prey taxa on the lower level are plotted using the Spearman's ρ correlation between their abundance and the land‐use intensity score, that is, positive values = taxa that increased in abundance with agricultural intensification. Link widths indicate the log_10_ frequencies with which taxa were consumed, coloured according to the comparison with the null model: red links = stronger than expected, blue links = weaker and grey = not significantly different. Node widths represent log_10_ mean abundance of each taxon: red nodes = preferred by both predators; blue nodes = avoided by both

**Figure 5:**
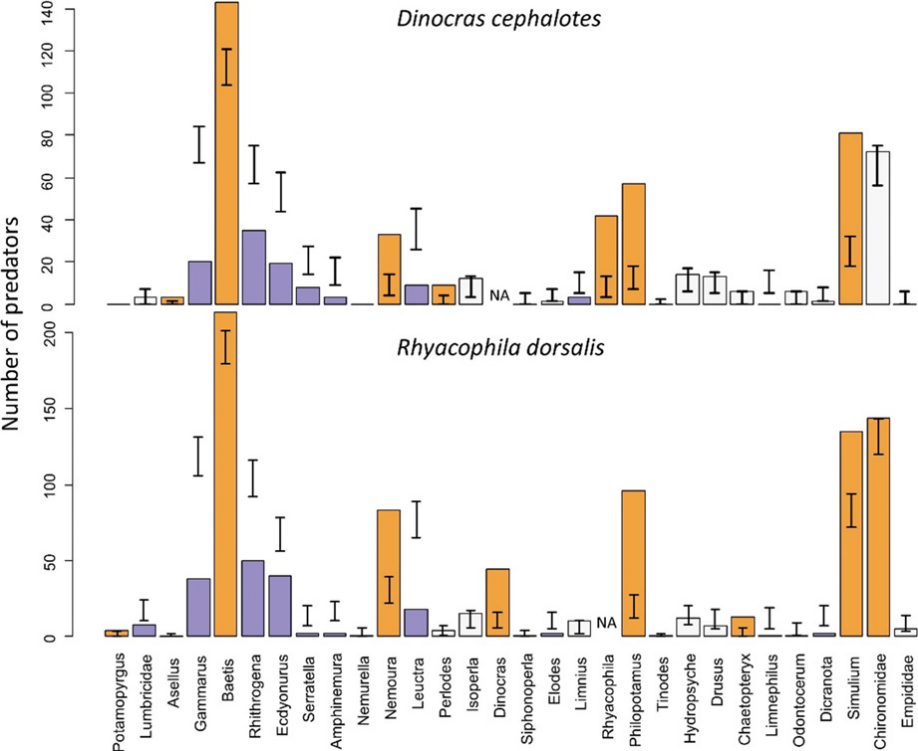
Number of predators consuming different prey taxa, compared to random expectation, based on prey availability. Error bars show 95% confidence limits of expected consumption: observed values falling outside of this range indicate significant deviation from random foraging (orange = preferred taxon, purple = avoided taxon)

The two predators' diets showed many similarities (Figures 3, 4, 5). The mean number of prey taxa consumed by individuals of both species was 4.5 (±0.02 *SE*), and the overall niche breadths were 0.17 (±0.01 *SE*) for *R. dorsalis* and 0.19 (±0.02 *SE*) for *D. cephalotes*. Neither measure differed significantly between the predators (number of prey taxa *t* = 0.25, *df* = 49, *p = *.80; *B*_*A*_ *t* = 1.29, *df* = 49, *p = *.20). There was a large overlap in the prey taxa consumed (Figure 3), producing greater niche overlap than expected by chance, both for the whole data set (Pianka's measure *O*_*jk*_ = 0.95, *p *<* *.001) and for the 21 site–season combinations with both predators present (*O*_*jk*_ = 0.40–0.91, all tests *p *<* *.05). The most common constituents of the predator's diets were *Baetis,* Simuliidae*,* Chironomiidae*, Philopotamus and Nemoura*. Taxa preferentially consumed by both predators were often those which increased in abundance with more intensive land use (e.g., *Simuliium, Nemoura*; Figure 4), whereas prey apparently avoided by both predators included those negatively correlated with intensification (e.g., *Ecdyonurus, Amphineura*; Figure 4). Overall prey selection strength was near‐identical for the two predators: 0.29 for *R. dorsalis* and 0.33 for *D. cephalotes*.

Despite many similarities, the overall dietary composition differed between the two predators (PERMANOVA *F* = 2.07, *df* = 59, *p = *.043), reflecting some differences in prey choice. *Rhyacophila* consumed more chironomids, *Philopotamus* and *Nemoura* relative to predictions from the null model, whilst *D. cephalotes* consumed *Asellus*, which was absent from *R. dorsalis* diet (Figure 5). There was frequent intraguild predation, with both predators preferentially consuming each other (Figures 4 and 5). Both predators selectively consumed *Baetis, Nemoura, Philopotamus* and Simuliidae, and avoided Heptageniidae (*Rhithrogena* and *Ecdyonurus*), *Gammarus, Leuctra* and *Limnius*.

There was little evidence of a change in *R. dorsalis's* feeding niche between streams with and without *D. cephalotes* present (Figure 3); diet overlapped almost entirely and this overlap was much greater than expected by chance (*O*_*jk*_ = 0.973, *p *<* *.0001). There was no significant difference in dietary specialization (*F* = 1.402, *df* = 28, *p = *.199), nor in the overall selection strength (*F* = 0.19, *df* = 28, *p = *.85), but the pattern of prey selection was different with *R. dorsalis* preferentially consuming Chironomidae, *Rhithrogena* and *Ecdyonurus* in sites with *D. cephalotes* present but not in sites without *D. cephalotes* (Appendix S4).

### 3.4 Effects of land use and season on *R. dorsalis* diet and foraging behaviour

There was little evidence of changes in the diet or foraging behaviour of *R. dorsalis* across the agricultural gradient. The number of prey species, and strength and identity of trophic interactions were similar between the extremes of the intensity gradient (three highest vs. three lowest intensity sites; number of species 4.2 vs. 3.8 *t* = 0.51, *df* = 162, *p = *.30; strength of interactions 0.54 vs. 0.89, *t* = 0.15, *df* = 28, *p = *.14; Appendix S5).

Generally, the contribution of each prey taxon to *R. dorsalis* diet reflected its abundance in the environment with *Baetis*, Chironomidae and Simuliidae accounting for the largest proportions of prey taxa consumed. There were some changes in diet with increasing intensity amongst the less abundant taxa, with sensitive species (e.g., *D. cephalotes*,* Siphonoperla* and *Amphinemura*) absent from high‐intensity sites and others (e.g., *Potamopyrgus*) absent from the lowest intensity sites (Appendix S5). The optimal model for overall strength of *R. dorsalis* prey selectivity contained only land‐use intensity but the relationship was not significant (*t* = 1.75, *df* = 8, *p = *.118, *R* ^2^ = .19, respectively).

For models of the effect of intensity and season on *R. dorsalis* number of prey taxa and dietary specialization (*B*_*A*_), the lowest AIC value was obtained when the season was the only predictor variable. Only diet breadth was significantly different between seasons. The number of prey taxa consumed per individual was significantly greater in June and September than December or February, respectively (*F* = 4.2, *df* = 1,9, *p = *.04 and *F* = 8.1, *df* = 1,9, *p = *.03).

## 4 DISCUSSION

Large invertebrate predators can exert top‐down control on communities such that their feeding habits can influence ecosystem functioning (Wipfli & Gregovich, 2002). Despite this, changes in predator feeding behaviour along stress gradients have received little attention. Here, in one of the first uses of molecular techniques to improve the resolution and accuracy of feeding interaction characterization, *R. dorsalis* and *D. cephalotes* were shown to be generalist predators, which preferentially consumed the most abundant prey taxa. Agricultural intensification did not significantly change predator foraging behaviour or diet, as preferred prey taxa were resistant to agricultural stressors and abundant across the intensity gradient. There was, however, a suggestion of community simplification at the highest intensities with the loss of *D. cephalotes* and several *R. dorsalis* prey items. Although the diet varied amongst seasons, the effects of agricultural intensification were consistent across them.

### 4.1 Evaluating the ion torrent sequencing approach for invertebrate diet analysis

Molecular techniques provide valuable tools for constructing empirical food webs, improving upon traditional techniques by increasing the detection of rare and soft‐bodied prey taxa, improving confidence in prey identification, and reducing processing time to allow larger sample sizes (=Wirta et al., 2014; Roslin & Majaneva, 2016). Sequencing results are, however, still subject to some of the same uncertainties present in the morphological gut content analysis, such as the inability to identify secondary predation (Sheppard et al., 2005) or scavenging (Symondson, 2002). Using NGS, the numbers of sequences amplified for each prey taxon cannot be used as a reliable guide to how many individuals, or even how much biomass, was consumed by each individual predator (Pompanon et al., 2012). Further, molecular sequencing has its own unique sources of error. The degree to which the technology used affects sequencing results remains uncertain, with MID choice, sequencing platform and MOTU clustering algorithm all potentially affecting results (Deagle, Thomas, Shaffer, Trites, & Jarman, 2013). Biases affecting numbers of prey sequences, and ways of calibrating these, are explored by Thomas, Jarman, Haman, Trites, and Deagle (2014) and Thomas (2015) and, in the light of the differences between results from the two sequencing runs in the present study, we recommend further work to quantify these uncertainties across a wider range of study systems. As there is no attempt made in this work to quantify predation on the basis of numbers of sequences, using instead the numbers of predators testing positive as a more conservative measure, the effects of sequencing run differences should be minimal.

In many systems, including the present study, the phylogenetic proximity of predator and prey prevents the use of predator blocking probes, presenting the risk that the majority of sequences will belong to the predator. This may reduce the ability to detect rare prey items (e.g., Piñol et al., 2014). Here, however, only 3% of usable sequences were the predator's own DNA. We attribute this to the ease of gut dissection in the relatively large predators used in this study and recommend sequencing without blocking probes for species where gut dissection is possible to ensure no loss of prey species.

Despite the uncertainties associated with sequencing of gut contents, the technology affords great potential to resolve trophic interactions and, as price per read falls, we anticipate that investigation of entire food webs will become easier. Yield‐effort curves showed that over 90% of the total number of taxa were detected from 67 individuals and over 50% were detectable from just nine individuals. This supports our selection of 10 individuals per site/season and demonstrates that the 250+ individuals sampled per species gave very high sampling completeness. Although some studies using visual gut content analysis have sampled over 1,000 predator individuals (Woodward & Hildrew, 2002), most studies are restricted to small sample sizes due to processing times (e.g., Masese et al., 2014; *n* = 61 individuals; Bo, Fenoglio, Malacarne, Pessino, & Sgariboldi, 2007 *n* = 60). In our study, a high degree of sampling completeness was achieved with relatively few predatory individuals. It would be valuable to compare this with communities containing more species‐rich prey assemblages to evaluate the utility of NGS.

The main prey taxa we identified are consistent with previous studies of the feeding behaviour of *R. dorsalis* and *D. cephalotes* based on visual gut content analysis. Muotka (1993) showed that *R. dorsalis* diets were dominated by simuliids, Baetidae and chironomidae, whilst Dudgeon (2000) and Bo et al. (2007) found predatory stonefly diets to be dominated by chironomidae, *Philopotamus* and Ephemeroptera. One of the main differences was in the prey diversity detected at the level of individual predators. Bo et al. (2007) found that *D. cephalotes* had only 1.13 (± 1.15) prey taxa per predator, from 15 taxonomic groups, compared to 4.5 prey taxa from 24 groups identified here. Although we cannot test this directly, this difference is consistent with the greater ability of molecular techniques to detect rare taxa and also prey during the entire duration of their passage through the gut (Symondson, 2002). With visual gut contents analysis, it can be very difficult to identify partially digested body fragments, especially for soft‐bodied taxa such as the oligochaetes and several dipteran larvae in our study. Further, studies using visual identification tend to group species at a high taxonomic level (e.g., order or family level), masking the consequences of changes in food web structure for functional diversity and wider ecosystem functioning. The taxonomic resolution in the present study was set mainly by the identification of the kick samples: molecular results identified the majority (71%) of taxa to species level, which may give greater insight into food web structure and is relevant where species have unique functional roles or are of conservation importance.

Two disadvantages of our molecular approach were the inability to resolve cannibalism and to identify vegetative material, which may make an important contribution to the diet of both predators, even at larger instars (Bo et al., 2007; Céréghino, 2002, 2006). We were unable to amplify DNA from the guts of around 20% of predators and suggest this may be because their guts contained only plant material. Future studies could screen in parallel with general plant primers (Haider, 2011; reviewed in Pompanon et al., 2012) to determine the level of herbivory.

### 4.2 Feeding behaviour and niche overlap between *R. dorsalis* and *D. cephalotes*

As predicted, and as observed by Dudgeon (2000) for predatory stoneflies, both predators appeared to consume prey approximately in proportion to their availability. Apparent prey choice was relatively modest and mainly reflected avoidance of larger prey taxa that were abundant in the community (e.g., heptageniids and *Gammarus*). These results suggest that several prey species offered nutritional equivalence and that encounter rate is likely to be the biggest determinant of prey choice for these predators, although capture success, handling efficiency and nutritional quality are also likely to play a role (Symondson, 2002). In addition, there was some evidence that the most abundant species were disproportionately consumed. This is consistent with optimal foraging theory which postulates that predators form a search image for the most common prey and increase the efficiency with which they capture and handle it, resulting in the most common prey becoming the most profitable for the predator (Krebs, Kacelnik, & Taylor, 1978).

Contrary to predictions, there was no significant change in *R. dorsalis* diets with increasing agricultural intensity. Despite significant changes in the invertebrate community across the intensity gradient, *R. dorsalis* mainly consumed prey taxa that were resistant to agricultural stressors and were present in all streams (Baetidae, Chironomidae, Simuliidae and *Philopotamus*). There was, however, a change amongst the rarer taxa in its diet, reflecting the replacement of taxa sensitive to agricultural stressors (e.g., *Siphonoperla*,* Amphinemura*) with others that were only present at high‐intensity sites (e.g., *Potamopyrgus*).

The largest effect of agricultural intensification for the present study was the loss of *D. cephalotes* from the highest intensity sites. The very high overlap in dietary niche and similar overall prey selection strength of the two predators suggests that *D. cephalotes* is not, as hypothesized, a more specialized predator than *R. dorsalis,* and therefore, its lower resilience to agricultural stressors was unlikely to be a result of feeding behaviour. Several of the prey taxa that were most heavily selected for by *D. cephalotes* (e.g., Baetidae, Simuliidae and *Philopotamus*) were present across the agricultural gradient, such that declines in prey availability could not explain the loss of *D. cephalotes* at high agricultural intensities. Instead, the loss of *D. cephalotes* seems likely to be more the results of direct sensitivity to physicochemical stressors, notably fine sediments (Turley et al., 2016), although changes in biotic interactions, such as competition with other predators, or a combination of abiotic and biotic factors (e.g., Cadotte & Tucker, 2017) cannot be ruled out as possible causes. Understanding the effects of losing a predator on community dynamics is critical for understanding the functional consequences of biodiversity loss (Worsfold, Warren, & Petchey, 2009). Here, the loss of *D. cephalotes* did not affect significantly the feeding niche, dietary specialization or overall prey selection strength of *R. dorsalis*, despite the high niche overlap between these predators suggesting they could be competitors. There was evidence of modest changes in prey preferences, however. Determining the effect of competition on feeding behaviour, and the wider consequences for the community, would require the whole food web to be resolved.

The generality of *D. cephalotes*'s feeding behaviour makes it unlikely that its loss would result in major changes to community structure (Worsfold et al., 2009), but it may be symptomatic of other changes that occurred in the food web. Previous work has demonstrated increases in food chain length and connectance with mild nutrient enrichment from agricultural intensification due to greater availability of basal energy resources (Jaarsma, De Boer, Townsend, Thompson, & Edwards, 1998; Riley, Townsend, Niyogi, Arbuckle, & Peacock, 2003; Townsend, Thompson, McIntosh, & Kilroy, 1998). The current study covered a longer intensity gradient, including much higher nutrient and sediment concentrations, than those previous studies. The declines in taxon richness in response to physicochemical stressors suggest as intensification increases further, an overall simplification of food web structure occurs.

Simplification of stream food webs has also been observed in response to acidification (Layer et al., 2010) and drought (Ledger et al., 2013). Both studies showed streams under stress to have smaller food webs with fewer trophic interactions. In theory, simplification may make food webs more stable if interaction strengths remained constant, but if the number and positioning of strong links is altered, the consequences for ecosystem stability and functioning could be far‐reaching (Ledger et al., 2013; McCann, 2000). Quantifying interaction strengths and site‐specific foraging behaviour is therefore a priority for food web studies. The present results provide a first indication of changes in trophic interactions over a wide stressor gradient, but further work is required to expand this to the food web and to determine the consequences for stability and ecosystem functioning.

In line with the positive association between algal productivity and food web size and connectance described by Townsend et al. (1998), the number of prey taxa consumed was higher in June and September than December or February. This result was also observed by Woodward, Speirs, and Hildrew (2005) and attributed to a higher abundance of rare prey items in summer months when in‐stream production was highest. Predator feeding behaviour was unchanged across seasons due to the generality of these predators and abundance of preferred prey taxa across the intensity gradient in all seasons.

Overall, this study was able to resolve the diets of both predatory taxa along a land‐use gradient with a high degree of replication and sampling completeness. It also demonstrated that sequencing without blocking probes on dissected predator guts is a successful method for determining stream invertebrate diets, with many potential advantages over traditional visual techniques. Enhanced resolution of trophic interactions will improve our understanding of the complex direct and indirect effects of anthropogenic stressors on ecosystem functioning (Gray et al., 2014). The consistency of predator feeding behaviour with increasing agricultural intensity observed here is a first step towards understanding the thresholds at which land‐use change may disrupt stream ecosystem functioning.

## DATA ACCESSIBILITY

All data sets are available through Dryad https://doi.org/10.5061/dryad.0hb5n.

## AUTHOR CONTRIBUTIONS

C.P., I.P.V., S.J.O. and W.O.C.S. designed the study. C.P. and E.I.B. performed all the laboratory work and data analysis, with guidance from W.O.C.S. and I.P.V., respectively. E.C. developed the technique for sequence analysis and guided C.P. in performing these. I.P.V. developed the techniques to analyse prey selectivity. All authors contributed to writing the manuscript.
