# Supplementary material for: The effects of pastoral intensification on the feeding interactions of generalist predators in streams
Source: Mol Ecol. 2017 Dec 23;27(2):590–602. doi: 10.1111/mec.14459 (PMC5887918; doi:10.1111/mec.14459)

The effects of pastoral intensification on the feeding interactions of generalist predators in streams

C.E. Pearson, W.O.C. Symondson, E.L. Clare, S.J. Ormerod, E. Iparraguirre Bolaños, and I.P. Vaughan

Supplementary Material

Appendix 1 – Deriving the intensity score.

Forty-five physico-chemical variables were recorded at each site in May 2012 to quantify the intensity of pastoral land use effects on streams (Table S1). Water chemistry was assessed by taking a 150 ml water sample from a riffle in opaque sterilised bottles (SciLabware, HDPE screw cap bottle). The sample was frozen on return to the laboratory and ionic concentrations were determined using ion chromatography for anions (Dionex DX-80 Ion Analyser; Thermo Fisher Scientific, Inc.) and inductively coupled plasma mass spectrometry for cations (Thermo Elemental X-Series ICP-MS; Thermo Fisher Scientific, Inc.). In addition, three measures of pH and conductivity were taken in each site using a handheld probe (HANNA instruments, pH/EC/TDS model HI98129).

Physical habitat was assessed over a 100 m reach (Table S1). Every 10 m the bank material, bank profile, predominant substrate, canopy cover and bank-top vegetation complexity were recorded following River Habitat Survey protocols (Environment Agency, 2003) and the width and depth measured at five equally spaced points across the channel. The total length of bank undergoing active fluvial erosion or livestock poaching was measured over the 100m reach. Flow velocity was measured at three locations in the fastest current and three locations in the margin over the area covered by the kick samples. Areal coverage of fine sediment (< 2 mm) was estimated in 5% increments within ten 0.25 m² quadrats which alternated between the channel centre and margin (Zweig and Rabeni, 2001). In addition, the amount of re-suspendable sediment was determined by pushing an open drum (25 cm diameter, 0.0625 m^2) into the substratum, disturbing the sediment to 2 cm depth for fifteen seconds and capturing a 300 ml sample (Larsen *et al.*, 2009). Three replicate samples were combined to create a 900 ml bulk sample from which sediment between 0.025 and 1 mm was filtered. Ash

free dry mass was then determined to distinguish organic and inorganic components (Riley *et al.*, 2003).

Table S1 – Description of physical habitat characteristics surveyed in 2012 and 2013.

Environmental variables	Units	Description
Depth	Metres (m)	Five measures across each of ten transects
Wetted width	Metres (m)	Measured at ten transects
Bank material	RHS categories	Every 10m: Boulder, Cobble, Gravel or Earth
Substrate	RHS categories	Every 10m: Bedrock, Boulder, Cobble, Pebble, Sand or Silt
Bank top vegetation	RHS categories	Every 10m: Complex, Simple, Uniform or Bare
Canopy cover	Absent, <33 %, >33 %, >66 %	Three transects within kick sample area
Bank profile	RHS categories	Every 10m: Vertical, Composite, Steep, Gentle
Conductivity	Parts per million (ppm)	Three replicates in kick sample area
Extent of fluvial erosion	Metres (m)	Length of bank undercut
Extent of bank poaching	Metres (m)	Length of bank slumping from livestock trampling
Flow velocity	Metres per second (ms^{-1})	Three replicates in mid-channel and three in margin
pH	None	Three replicates in kick sample area
Resuspendable sediment	Grams per litre	Weight of resuspendable inorganics (25µm to 1 mm) (May 2012 only)
Fine sediment cover	Percent (%)	Average fine sediment cover from 10 quadrats in mid-channel and margin
Water chemistry	mg L^{-1}	Flouride, Chloride, Nitrite, Bromide, Nitrate, Phosphate, Sulphate, Sodium, Magnesium, Aluminium, Potassium, Calcium, Vanadium, Chromium, Manganese, Iron, Copper, Nickle, Cobalt, Zinc, Arsenic, Cadmium, Lead and Selenium

A principal component analysis (PCA) was performed on the 45 physico-chemical variables to create the intensity score. The first principal component (PC1) explained 29.7 % of total variance and was highly correlated with percentage of the catchment under improved pasture (Pearson's r = 0.82). The variables with the strongest loadings on this axis were consistent with the expected effects of intensification (*e.g.* nitrate concentration, extent of bank poaching, fine sediment cover; Table S2). All other components explained < 10% of the variation and

were not related to agricultural intensity. PC1 scores were therefore interpreted as measure of pastoral intensification (‘intensity score’).

Table S2: Loading coefficients for Axis 1 of Principal Components Analysis of physico-chemical variables.

**Variable**	**Axis 1**
Calcium	0.25
Conductivity	0.24
Vanadium	0.24
Sulphate	0.24
Potassium	0.23
Sodium	0.23
Chloride	0.22
Magnesium	0.22
Nitrate	0.22
Chromium	0.21
Canopy cover	0.21
Length of bank poaching	0.20
Inorganic sediment	0.18
Organic sediment	0.18
Fine sediment cover	0.17
Bed sediment calibre	0.17
Extent of all erosion	0.17
Bank material calibre	0.15
pH	0.15
Gentle banks	0.14
Selenium	0.14
Artificial substrate	0.14
Iron	0.09
Nickel	0.09
Average width	0.05
Average mid-stream flow veloc	0.03
Cobalt	0.01
Vertical banks	0.01
Fluoride	-0.01
Average flow velocity at margin	-0.02
Bromide	-0.02
Phosphorus	-0.03
Steep banks	-0.03
Manganese	-0.04
Bedrock substrate	-0.04
Zinc	-0.05
Arsenic	-0.05
Undercut banks	-0.06
Bank vegetation score	-0.07

Composite banks	-0.09
Aluminium	-0.09
Copper	-0.10
Average depth	-0.12
Lead	-0.15
Cadmium	-0.15

Appendix 2 – Genera used to test generality of LCO-1490 and HCO-1777 primer pair for potential prey species.

Group represented	Family/ Genera tested
Target Predators	*Dinocras*
	Rhyacophila
Trichoptera	*Hydropysche*
Plecoptera	*Isoperla*
Ephemoptera	*Baetis*
Gastropods	Ancylus
	Physidae
Bivalvia	*Pisidium*
Amphipods	*Asellus*
Isopods	*Gammarus*
Diptera	Chironomidae
Tricladia	Planaria
Elmidae	*Limnius*
Hirudinaeta	*Eropobdella*
Lumbricidae	Oligochaetes
Scritidae	Aquatic beetles

Appendix 3 – Number of individual predator gut contents successfully sequenced from each survey site with sites labelled 1 to 10 from lowest agricultural intensity to highest agricultural intensity.

Site	***Dinocras cephalotes***				***Rhyacophila dorsalis.***				**Total**
	**Feb**	**June**	**Sep**	**Dec**	**Feb**	**June**	**Sep**	**Dec**	
1	8	8	8	10	3	10	2	6	55
2	2	4	6	5	4	2	3	9	35
3	6	8	13	10	3	4	0	3	47
4	9	6	7	10	7	2	1	8	50
5	0	0	5	8	14	4	12	8	51
6	4	6	6	8	6	10	0	9	25
7	0	0	0	0	7	8	10	1	50
8	0	0	0	0	11	9	7	11	38
9	0	0	0	0	6	8	2	9	25
10	0	0	0	0	2	7	2	7	18
Total	29	32	45	51	63	64	39	71	394

Appendix 4 - Selectivity graphs for Rhyacophila dorsalis in sites with (n=6) and without (n=4) Dinocras cephalotes present.

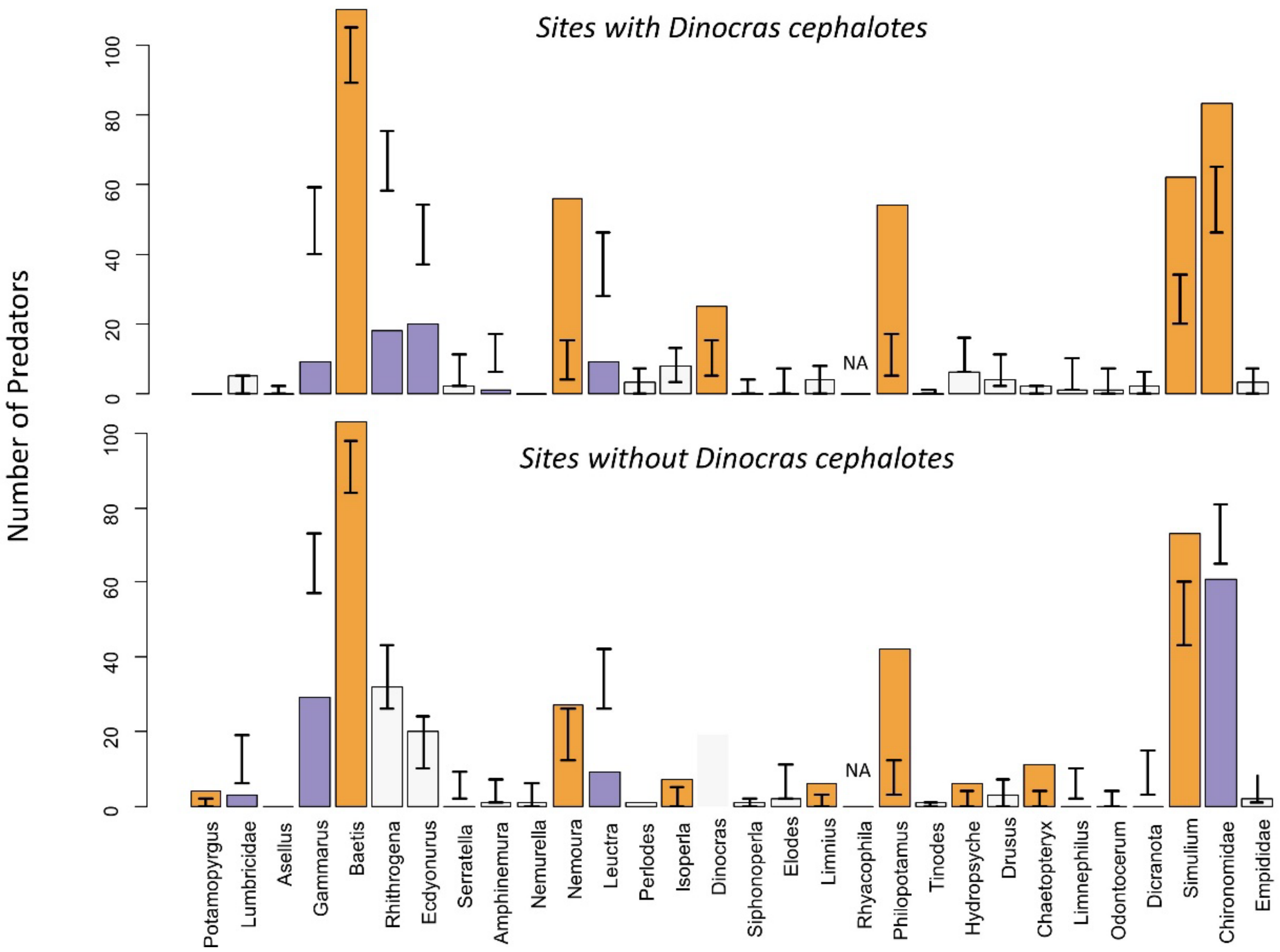

Appendix 5 - Selectivity graphs for Rhyacophila dorsalis in the three sites with the highest and lowest agricultural intensity.

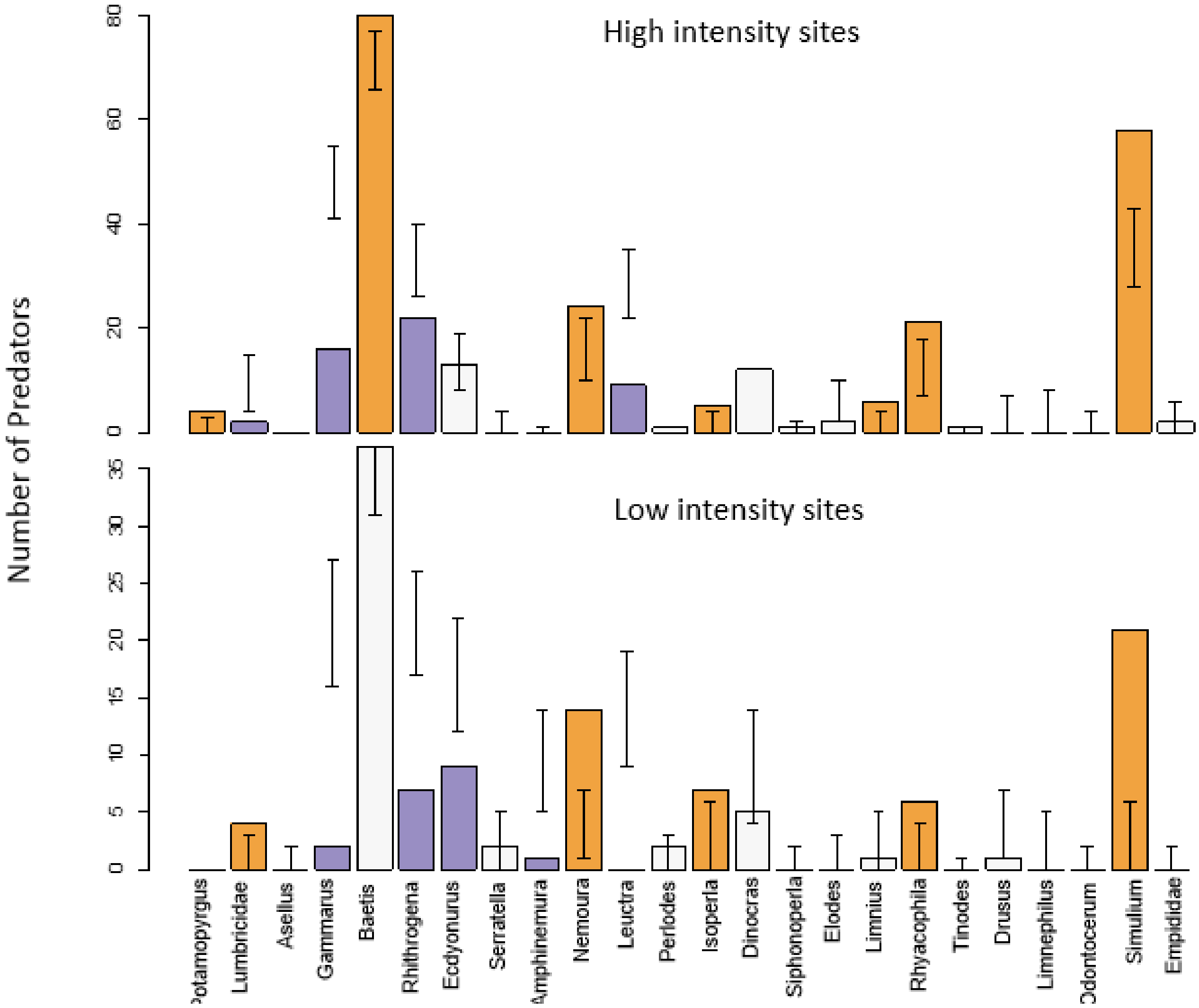

Supplement: Supplementary file 1 [file MEC-27-590-s001.pdf]
